# Gelatinized-Core and Chitosan-Coated Liposomes: A Stabilized Carrier for Glutathione Delivery

**DOI:** 10.3390/foods15213836

**Published:** 2026-10-27

**Authors:** Mihwa Hwang, Youjin Baek, Hyeon Gyu Lee, Do-Yeong Kim

**Affiliations:** 1 Department of Food and Nutrition, Hanyang University, 222, Wangsimni-ro, Seongdong-gu, Seoul 04763, Republic of Korea; 2 Korea Advanced Food Research Institute, 50, Botdeul-ro, Uiwang-si 16001, Republic of Korea; 3 Division of Food and Nutrition, Chonnam National University, 77, Yongbong-ro, Buk-gu, Jeonnam-Gwangju Special Metropolitan City 61186, Republic of Korea

**Keywords:** glutathione, chitosan coating, gelatinized-core liposomes, oxidative stability, GSH retention, cellular uptake

## Abstract

Glutathione (GSH) is a bioactive tripeptide with potent antioxidant activity, but its oral delivery is limited by gastrointestinal instability and low membrane permeability. This study developed a biopolymer-stabilized liposomal system by combining gelatin incorporation into the aqueous core with low-molecular-weight chitosan (CS) coating to improve GSH retention and delivery characteristics. Conventional liposomes (GSH/CL), gelatinized-core liposomes (GSH/GL), and CS-coated gelatinized-core liposomes (CS-GSH/GL) were prepared and comparatively evaluated. GSH/GL exhibited higher entrapment efficiency than GSH/CL, with further enhancement following CS coating. The CS-GSH/GL formulation with 1.6 mg/mL CS was selected as the optimal formulation based on its overall physicochemical properties. CS-GSH/GL also exhibited improved retention of thiol groups under oxidative and acidic conditions and maintained a stable dispersion under the tested pH conditions. Under simulated gastrointestinal conditions, CS-GSH/GL showed the lowest cumulative GSH release among the liposomal formulations, indicating improved retention of encapsulated GSH. In Caco-2 cells, the CS-coated gelatinized-core formulation showed the highest fluorescence intensity when rhodamine B, a fluorescent model compound, was used. These findings indicate that the combined core gelatinization and CS coating improved the physicochemical stability and GSH retention of liposomes, while enhancing the cellular association of the rhodamine B-loaded liposomal delivery system. Overall, these in vitro findings support the potential of the formulation for GSH protection and retention, although further in vivo studies are needed to evaluate its bioavailability and safety.

## 1. Introduction

Glutathione (ɣ-glutamyl-cysteinyl-glycine; GSH) is an endogenous tripeptide that plays a central role in cellular redox regulation and antioxidant defense [1]. Owing to the thiol (-SH) group of its cysteine residue, GSH effectively scavenges reactive oxygen species and maintains intracellular redox homeostasis [2]. In addition to its antioxidant activity, GSH has been associated with anti-inflammatory, detoxifying, and protective physiological functions [3]. However, the oral delivery of GSH remains challenging because of its susceptibility to gastrointestinal degradation, limited membrane permeability, and physicochemical instability caused by thiol oxidation [4,5].

To overcome these limitations, various nanoencapsulation systems have been investigated to improve the stability and bioavailability of GSH [6,7]. Among them, liposomes are particularly attractive because of their biocompatibility, structural similarity to biological membranes, and ability to protect encapsulated compounds from environmental degradation [8]. Nevertheless, conventional liposomes often exhibit low encapsulation efficiency for hydrophilic compounds and limited stability under adverse environmental conditions [9].

Recently, biopolymer-integrated liposomal systems have attracted increasing attention as advanced delivery platforms. Various biopolymers, including N,N-dimethylhexadecyl carboxymethyl chitosan [10,11], β- cyclodextrin [12], and proteins such as casein [13] and β-lactoglobulin [14], have been investigated for their potential to improve the physicochemical properties and stability of liposomal systems. Gelatin offers several advantages, including cost-effectiveness, ease of production, biocompatibility, and biodegradability [15]. Gelatinized-core liposomes incorporate gelatin into the internal aqueous phase to form a gel network, thereby enhancing encapsulation efficiency and structural stability through increased core viscosity [16,17]. Although gelatinized-core liposomes have shown advantages over conventional liposomes, gelatin networks remain vulnerable to acidic conditions, which may compromise structural integrity and promote leakage of encapsulated compounds [18]. Therefore, additional stabilization strategies are required.

Surface modification using cationic biopolymers has emerged as an effective approach to enhance liposomal stability and functionality [19]. Chitosan coating can improve membrane stability, cellular interaction, and structural integrity through electrostatic and hydrogen-bonding interactions [20]. In particular, low-molecular-weight chitosan (CS) exhibits improved solubility and processability while retaining the beneficial properties of chitosan [21]. Previous studies have demonstrated that the molecular weight of chitosan affects its interaction with liposomal membranes, coating characteristics, physicochemical stability, and cellular transport. For example, chitosan with different molecular weights exhibited distinct coating behaviors and binding efficiencies on liposomes, while low-molecular-weight chitosan showed enhanced transport across Caco-2 cell layers compared with high-molecular-weight chitosan [22,23]. In addition, chitosan has been evaluated in human studies for its safety and functional effects, supporting its use as a food-grade material for oral delivery applications [22]. Although CS coating has been reported to improve liposomal stability, encapsulation efficiency, and permeability [16], its stabilizing effects on gelatinized-core liposomes have not yet been systematically investigated, particularly in relation to the stability and delivery characteristics of GSH-loaded systems.

Therefore, this study aimed to develop a biopolymer-stabilized liposomal delivery system by integrating gelatinized core structuring and CS surface coating to enhance the physicochemical stability and delivery performance of GSH. GSH-loaded conventional liposomes (GSH/CL), gelatinized-core liposomes (GSH/GL), and CS-coated gelatinized-core liposomes (CS-GSH/GL) were prepared and comparatively evaluated in terms of their physicochemical properties, stability, release behavior, cytotoxicity, and cellular uptake.

## 2. Materials and Methods

### 2.1. Materials

GSH (reduced), L-α-phosphatidylcholine, gelatin type B from bovine skin (75 bloom), mucin from porcine stomach, L-cysteine hydrochloride, 5,5′-Dithiobis 2-nitrobenzoic (DTNB), 3-(4,5-dimethylthiazol-2-yl)-2-5-diphenyltetrazolium bromide (MTT), 4′, 6-diamidino-2-phenylindole (DAPI), rhodamine B sodium salt, and 10% formalin solution were purchased from Sigma-Aldrich Chemical Co. (St. Louis, MO, USA). Cholesterol was obtained from Yakuri Pure Chemicals Co. (Kyoto, Japan). Chitosan (degree of deacetylation, 87%; molecular weight, 2.5 kDa) was supplied from Kittolife Co. (Seoul, Republic of Korea). Methanol and water were of HPLC grade, and all other reagents were of analytical grade. The human colon carcinoma cell line (Caco-2) was supplied from the Korean Cell Line Bank (Seoul, Republic of Korea). Minimum essential media (MEM), MEM non-essential amino acids (MEM-NEAA), penicillin-streptomycin solutions, 0.25% trypsin-EDTA solution (1×), Dulbecco’s phosphate-buffered saline (DPBS), and phosphate-buffered saline (PBS) were obtained from Welgene Inc. (Gyeongsan, Republic of Korea). Fetal bovine serum (FBS) was procured from AbFrontier Co., Ltd. (Seoul, Republic of Korea).

### 2.2. GSH Quantification

GSH was quantified using high-performance liquid chromatography (HPLC, Waters Corporation, Milford, MA, USA) on a system consisting of a Waters 1527 binary pump, Waters 2487 dual λ absorbance detector, and a Zorbax Eclipse XDB-C18 column (250 × 4.6 mm, 5 µm). The mobile phase consisted of a mixture of 20 mM phosphate buffer (pH 2.5) and methanol (95:5 *v*/*v*) at a flow rate of 0.8 mL/min. A 10 µL injection volume was employed, and detection was carried out at a UV wavelength of 200 nm.

### 2.3. Preparation of GSH-Loaded Liposomes

GSH/CL, GSH/GL, and CS-GSH/GL were prepared using a modified thin-film hydration method [24]. Briefly, L-α-phosphatidylcholine and cholesterol were mixed in a 10:1 (*w*/*w*) ratio and dissolved in 99.9% ethanol. The mixture was subjected to a rotary evaporator (EYELA, Tokyo, Japan) at 50 °C for 15 min under reduced pressure to remove the organic solvent. The lipid film, formed in a round flask, was hydrated with GSH solution dissolved in either water or 2.5 mg/mL gelatin solution at 50 °C through stirring without vacuum for 30 min. The resulting suspensions were further stirred at room temperature for 1 h to form a robust membrane. Subsequently, the prepared liposomes were ultrasonicated at 30% amplitude for 3 min (VC 505, Vibracell Sonics, Newton, MA, USA) in an ice bath. CS-GSH/GL was prepared with a dropwise method. CS solutions were prepared with various concentrations (0.8, 1.2, 1.6, and 2.0 mg/mL). Under magnetic stirring at 1000 rpm, the uncoated GSH/GL was added dropwise into an equivalent volume of CS, followed by continued magnetic stirring for 20 min.

### 2.4. Characterization of GSH-Loaded Liposomes

Particle size, polydispersity index (PDI), and zeta potential of the samples were measured with Zetasizer Nano ZS (Malvern Instruments Ltd., Malvern, UK) by dynamic light scattering (DLS) method at 25 ± 1 °C.

### 2.5. Entrapment Efficiency

Entrapment efficiency was determined using a modified dialysis method [25]. Liposomal suspension (2 mL) was loaded into dialysis tubing (MW 12–14 kDa) and dialyzed against 100 mL distilled water at 100 rpm for 4 h, with the external medium replaced every hour. After dialysis, the retained suspension was diluted with ethanol and centrifuged at 17,000 rpm for 20 min. Encapsulated GSH was quantified by HPLC, and entrapment efficiency was calculated using Equation (1):

(1) $$\mathrm{Entrapment}\;\mathrm{efficiency}\;(\%)=\frac{\mathrm{Amount}\;\mathrm{of}\;\mathrm{GSH}\;\mathrm{encapsulated}\;\mathrm{in}\;\mathrm{vesicle}}{\mathrm{Total}\;\mathrm{amount}\;\mathrm{of}\;\mathrm{GSH}}\times 100$$

### 2.6. Stability

#### 2.6.1. Oxidative Stability

Oxidative stability was evaluated by determining thiol group content following H_2_O_2_ treatment. Samples were oxidized with 10 µL of 3% H_2_O_2_ and analyzed using Ellman’s assay [6]. The 3% H_2_O_2_ solution (10 µL) was added to 100 µL of sample, corresponding to an H_2_O_2_ solution-to-sample volume ratio of 1:10. Briefly, 1 mL of 0.1 M sodium phosphate buffer, 20 µL of DTNB (4 mg/mL in 0.1 M sodium phosphate buffer of pH 8.0), and 100 µL of the oxidized samples underwent incubation at room temperature for 15 min. Subsequently, the absorbance was measured at 412 nm using the Synergy HT multi-microplate reader. Thiol group quantification was performed using L-cysteine hydrochloride as a standard. Thiol-group content was selected as an indicator of stability because the free sulfhydryl group of GSH is directly involved in its redox activity. Ellman’s assay was therefore used to compare the preservation of reduced thiol functionality among formulations under oxidative and different pH conditions.

#### 2.6.2. pH Stability

The pH of free GSH and GSH-loaded liposomes was adjusted to 1, 3, 5, and 7 using either 1 M HCl or NaOH and incubated at 37 °C for 2 h. Stability was evaluated by determining thiol group content using the Ellman assay as described above.

### 2.7. In Vitro Release Property

The in vitro release properties of both free GSH and GSH-loaded liposomes were determined through a dialysis method within simulated gastrointestinal environments [26]. Simulated gastric fluid (SGF) was formulated by dissolving 2 g of NaCl and 3.5 mL of 2 M HCl in 1 L of distilled water, with pH adjusted to 1.2 using 2 M HCl. Simulated intestinal fluid (SIF) was prepared by dissolving 6.8 g of KH_2_PO_4_ and 0.616 g of NaOH in 1 L of distilled water, with pH adjusted to pH 6.8 with 2 M NaOH. Enzyme-free SGF and SIF were used to compare the release behavior of free GSH and GSH-loaded liposomes under pH conditions representative of the gastric and intestinal environments. Subsequently, 5 mL of the prepared free GSH and GSH-loaded liposomes were introduced into dialysis tubing. The tubing was immersed in 85 mL of SGF and stirred at 100 rpm at 37 °C for 2 h, followed by transfer into 85 mL of SIF for an additional 2–12 h. Sampling occurred at predetermined intervals of 0.5, 1, 1.5, and 2 h for SGF and 3, 4, 6, 8, and 12 h for SIF. At each time point, 1 mL of dissolution medium was collected, and an equal volume of fresh medium was introduced. The quantification of released GSH was conducted using HPLC, as described in Section 2.2.

### 2.8. Cytotoxicity

Cytotoxicity of free GSH and GSH-loaded liposomes was assessed using the MTT assay [27]. Caco-2 cells were seeded in 96-well plates (1.0 × 10^5^ cells/mL) and incubated at 37 °C with 5% CO_2_ for 24 h. Caco-2 cells were cultured in minimum essential medium (MEM) supplemented with 45% (*v*/*v*) fetal bovine serum (FBS), 4.5% (*v*/*v*) penicillin–streptomycin solution, and 0.5% (*v*/*v*) MEM non-essential amino acid solution. Cells were treated with free GSH or GSH-loaded liposomes (10–80 µg/mL GSH equivalent) for 24 h. After incubation with MTT solution (5 mg/mL in PBS) for 4 h, the plate was centrifuged at 1500 rpm for 10 min, and the supernatant was removed. Subsequently, 150 µL of dimethyl sulfoxide (DMSO) was added to each well to dissolve the formazan crystals, followed by incubation for 15 min. The absorbance was measured at 540 nm using a Synergy HT Multi-Mode Microplate Reader. Cell viability was calculated using Equation (2):

(2) Cell viability (%) = [(A_sample − A_blank)/A_control] × 100

where A_sample, A_blank, and A_control represent the absorbance values of the sample, blank, and control, respectively.

### 2.9. Cellular Uptake

Cellular uptake of the liposomal formulations was qualitatively evaluated in Caco-2 cells using confocal laser scanning microscopy with rhodamine B (Rho) as a fluorescent model compound [28]. Caco-2 cells were seeded in a 6-well plate (4.0 × 10^5^ cells/mL) and incubated at 37 °C with 5% CO_2_ for 24 h. Free Rho, Rho-loaded conventional liposomes (Rho/CL), Rho-loaded gelatinized-core liposomes (Rho/GL), and CS-coated Rho-loaded gelatinized-core liposomes (CS-Rho/GL) were added to the cells and incubated at 37 °C with shaking. After 6 h, the medium was removed and the cells were washed twice with ice-cold PBS. The cells were then fixed with 10% formalin for 20 min, followed by DAPI staining for 20 min in the dark. After staining, the cells were washed twice with PBS, and the coverslips were mounted onto glass slides. Fluorescence images were obtained using a confocal microscope (STELLARIS8 STED & FALCON, Leica, Mannheim, Germany). The excitation/emission wavelengths were 543/593 nm for Rho and 401/421 nm for DAPI. Intracellular fluorescence intensity was quantified using LAS X software (version 3.7.5.24914, Leica Microsystems, Wetzlar, Germany).

### 2.10. Statistical Analysis

All experiments were performed at least in triplicate. Data are expressed as the mean value ± standard deviation (SD). Physical properties were analyzed using Student’s *t*-test, whereas other data were analyzed by one-way ANOVA followed by Duncan’s multiple range test using SPSS version 27.0 (SPSS Inc., Chicago, IL, USA). Differences were considered significant at *p* < 0.05.

## 3. Results and Discussion

### 3.1. Physical Properties of GSH-Loaded Liposomes

The physical characteristics of GSH/CL and GSH/GL are presented in Table 1. The gelatin concentration was fixed at 2.5 mg/mL based on a previous study [29]. GSH/CL showed a particle size of 100 ± 2 nm, a PDI of 0.260 ± 0.016, and a zeta potential of −55 ± 4 mV, whereas GSH/GL exhibited a significantly smaller particle size of 92 ± 2 nm and a less negative zeta potential of −22 ± 1 mV (*p* < 0.05). Entrapment efficiency also increased significantly from 34 ± 6% to 47 ± 3% after core gelatinization (*p* < 0.05). Both formulations showed PDI values below 0.3, indicating a relatively uniform size distribution [30]. The decrease in particle size following core gelatinization may be associated with electrostatic interactions between positively charged gelatin and negatively charged regions of the liposomal membrane, which have been suggested to increase vesicle rigidity and compactness [16,31]. In a previous study, TEM images of conventional and gelatinized-core liposomes containing EGCG revealed spherical morphology and a unilamellar structure, with morphological differences observed following gelatin incorporation [29]. The increased entrapment efficiency of GSH/GL may be attributed to the increased viscosity of the internal aqueous phase upon gelatin incorporation, which may help reduce GSH leakage during preparation [32,33]. Although the zeta potential of GSH/GL shifted toward less negative values, the formulation remained below the level generally considered sufficient for strong electrostatic stability.

**Figure 1:**
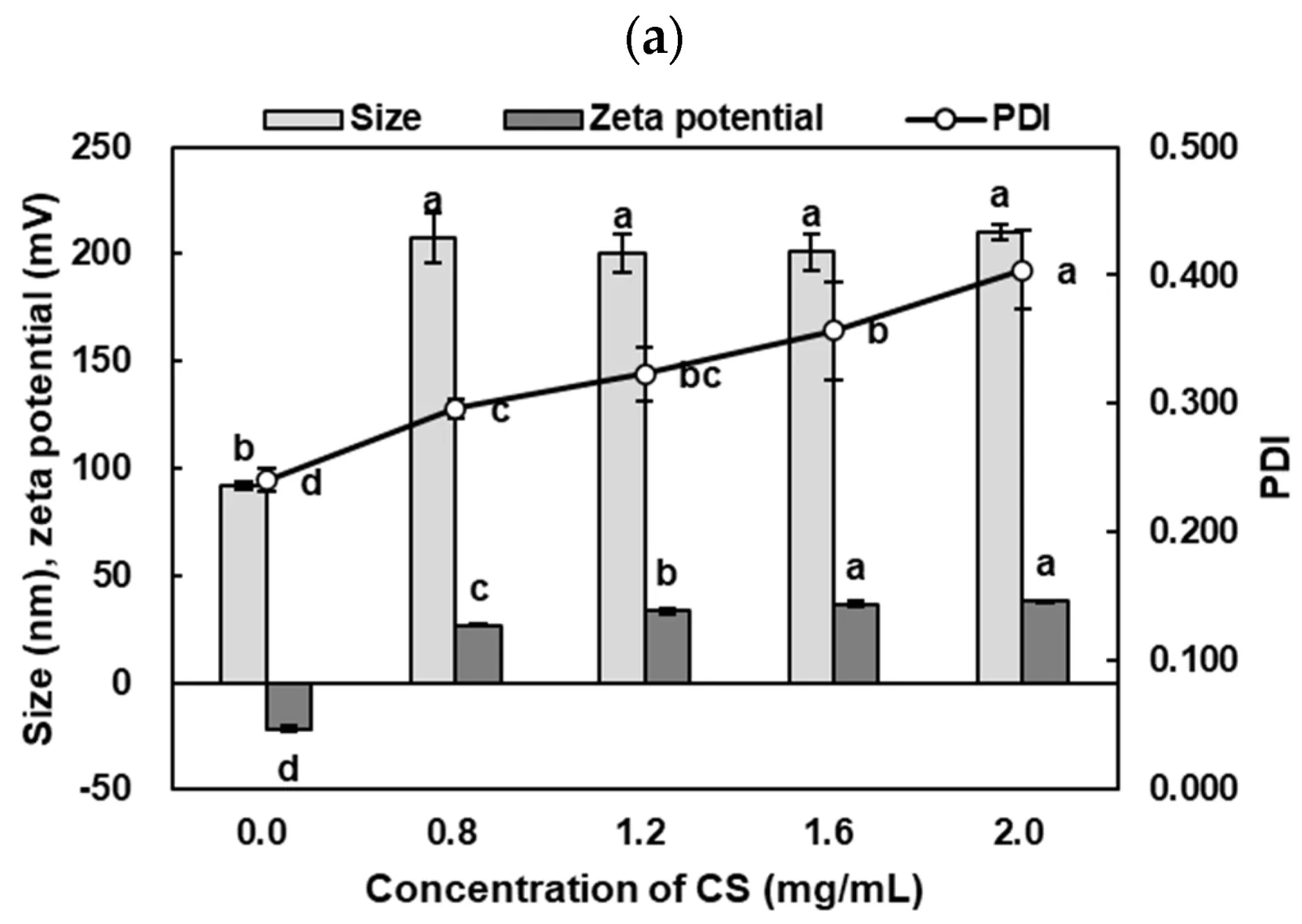

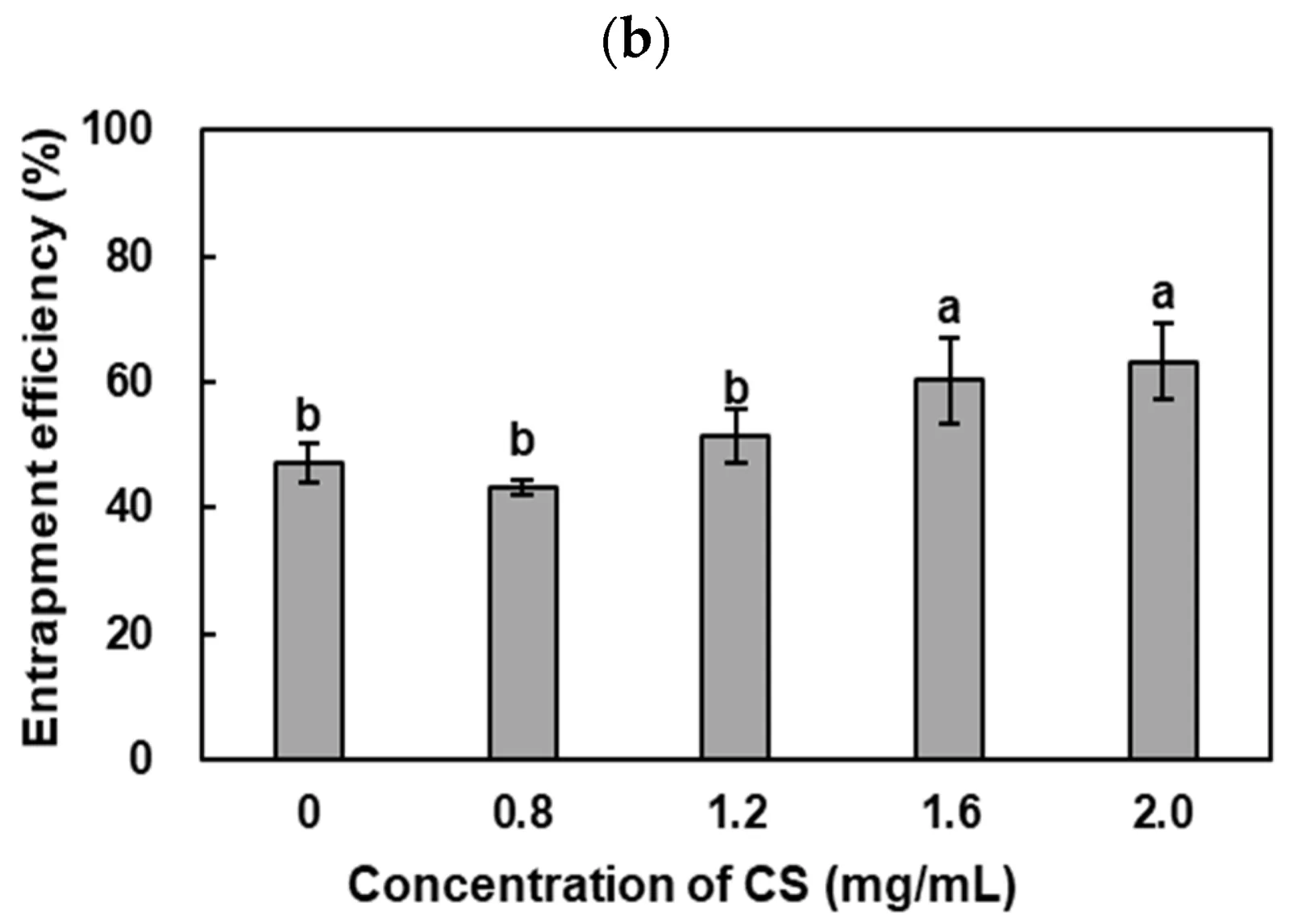
Effect of CS coating concentration (0, 0.8, 1.2, 1.6, and 2.0 mg/mL) on (**a**) particle size, PDI, and zeta potential, and (**b**) entrapment efficiency of CS-GSH/GL. Different lowercase letters represent significant differences (*p* < 0.05). Data are expressed as mean ± SD.

The effects of CS coating on the physicochemical properties of liposomes are shown in Figure 1. Preliminary observations indicated that CS concentrations below 0.8 mg/mL resulted in large aggregates and phase separation, whereas concentrations above 2.0 mg/mL led to particle sizes exceeding 250 nm and PDI values above 0.5.; therefore, CS concentrations ranging from 0.8 to 2.0 mg/mL were evaluated. CS coating significantly increased particle size from 92 ± 2 nm to approximately 200–210 nm (*p* < 0.05), consistent with the formation of a polymer-associated surface layer. No significant differences in particle size were observed among CS concentrations. In contrast, PDI increased significantly with increasing CS concentration, reaching 0.404 ± 0.030 at the highest concentration (*p* < 0.05). Previous studies have reported that excessive chitosan concentrations may induce bridging flocculation through interactions between extended chitosan chains on adjacent liposome surfaces, potentially compromising dispersion stability during storage [34,35]. Thus, an appropriate chitosan concentration is essential to ensure sufficient surface coverage while minimizing polymer-mediated aggregation. Accordingly, a CS concentration of 1.6 mg/mL was selected to achieve improved entrapment efficiency while limiting the increase in PDI, which could potentially affect long-term storage stability.

Zeta potential shifted from −22 ± 1 mV to positive values ranging from 27 ± 1 to 38 ± 1 mV in a concentration-dependent manner (*p* < 0.05), supporting the association of positively charged CS with the liposomal surface.

Entrapment efficiency increased from 47 ± 3% to 63 ± 6%, with a significant increase observed at CS concentrations ≥1.6 mg/mL (*p* < 0.05). The increased entrapment efficiency may be associated with the additional surface barrier provided by CS, which could reduce GSH diffusion from the liposomal structure. Based on these results, 1.6 mg/mL was selected as the optimal CS concentration because it provided improved electrostatic stability and encapsulation efficiency while maintaining acceptable particle size and dispersity.

### 3.2. Oxidative Stability

**Figure 2:**
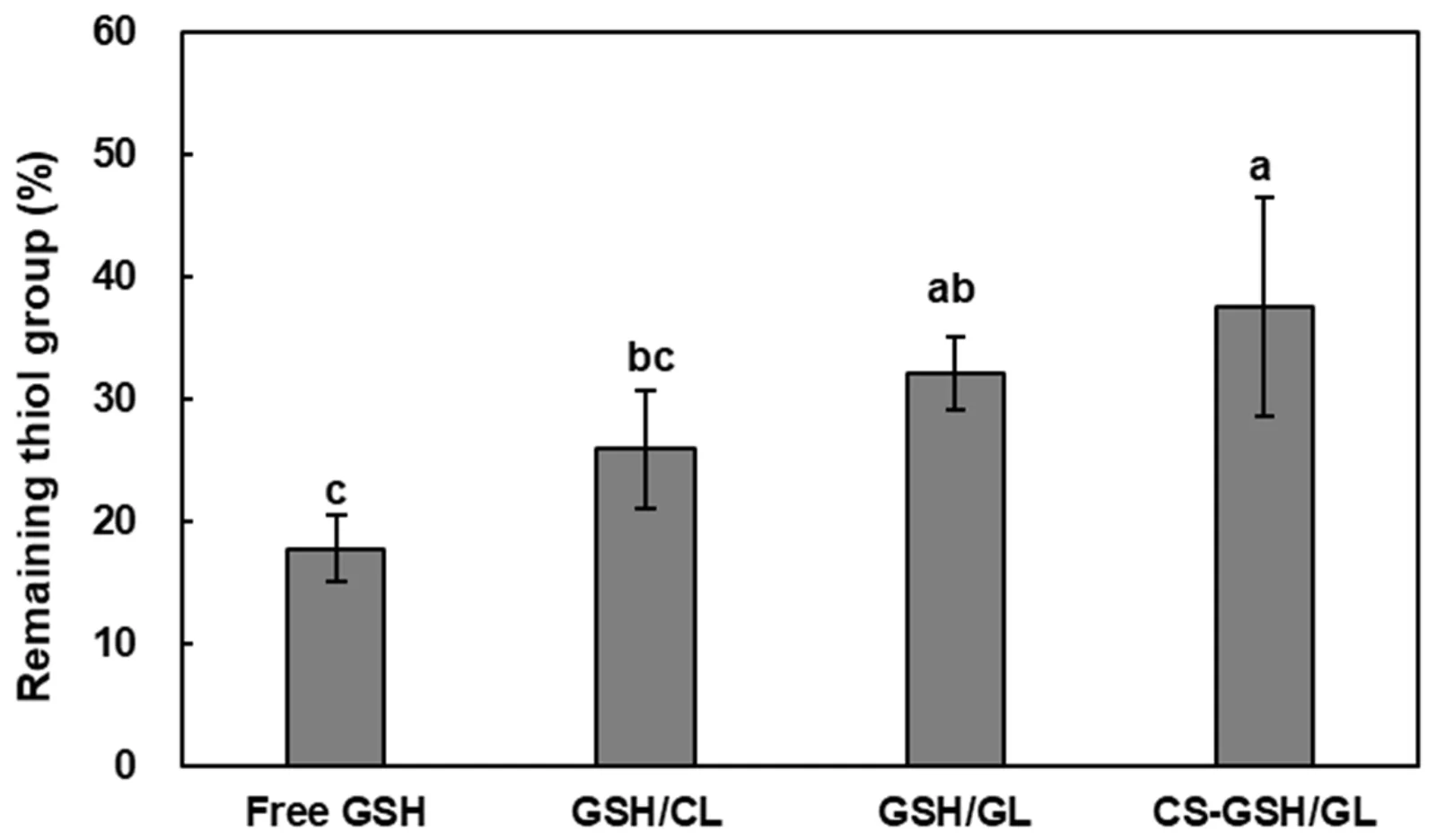
Effect of oxidative treatment on the thiol group content of free GSH, GSH/CL, GSH/GL, and CS-GSH/GL. Different lowercase letters indicate significant differences (*p* < 0.05). Data are expressed as mean ± SD.

The stability of thiol groups in free GSH and GSH-loaded liposomes under oxidative stress is presented in Figure 2. Thiol group contents decreased in all samples after H_2_O_2_ treatment, but the liposomal formulations retained significantly higher thiol levels than free GSH. Among the formulations, CS-GSH/GL exhibited the highest thiol content, corresponding to approximately a 2.1-fold improvement in oxidative stability compared with free GSH (*p* < 0.05). The reduction in thiol content after exposure to 3% H_2_O_2_ is attributed to the oxidation of sulfhydryl groups to the corresponding oxidized form [6].

Free GSH showed the greatest reduction in thiol content, followed by GSH/CL, GSH/GL, and CS-GSH/GL. The relatively greater loss observed for GSH/CL may be associated with the limited barrier provided by the conventional liposomal structure under oxidative conditions [36]. Gelatin incorporation and subsequent CS coating were associated with greater retention of thiol groups, which may reflect improved structural protection of encapsulated GSH and reduced exposure to the external oxidative environment [37,38]. Overall, the results indicate that the modified liposomal structures provided greater protection of GSH thiol groups than free GSH under the oxidative conditions tested.

### 3.3. pH Stability

**Figure 3:**
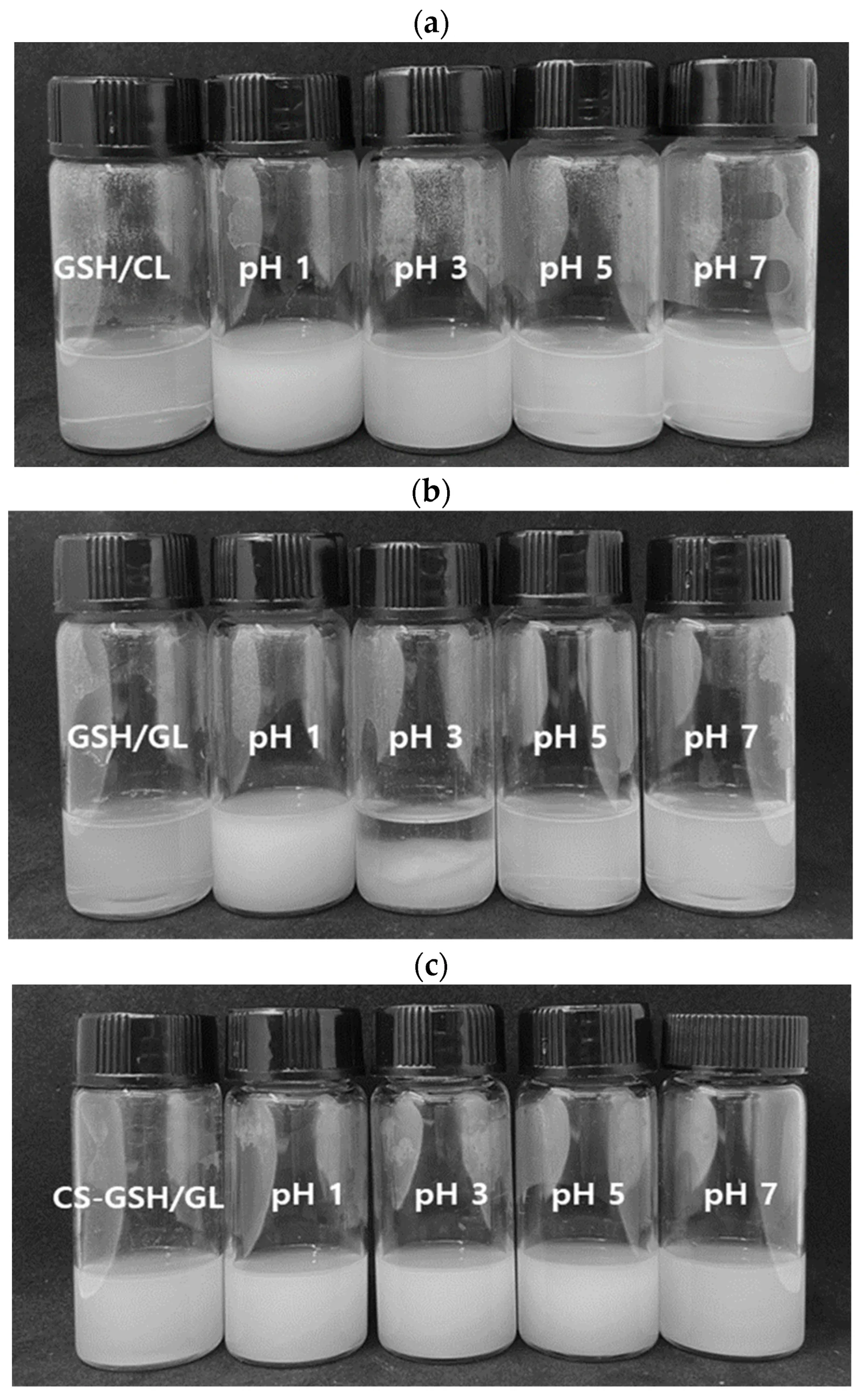
Visual appearance of GSH/CL (**a**), GSH/GL (**b**), and CS-GSH/GL (**c**) at different pH values.

Maintaining the physical stability of GSH-loaded liposomes under different pH conditions is relevant to their behavior during oral delivery. The visual appearance of GSH/CL, GSH/GL, and CS-GSH/GL at pH 1, 3, 5, and 7 is presented in Figure 3. As shown in Figure 3a,b, turbidity increased under strongly acidic conditions (pH 1 and pH 3), and GSH/GL exhibited precipitation at pH 3. In contrast, CS-GSH/GL maintained stable dispersion without visible precipitation across all tested pH conditions (Figure 3c).

Liposome dispersion stability is strongly influenced by environmental pH, with acidic conditions promoting membrane destabilization and aggregation due to hydrogen ion-induced structural disruption [26,39,40]. The precipitation of GSH/GL at pH 3, but not at pH 1, suggests that visible precipitation was not governed solely by increasing acidity. Previous research has shown that gelatin self-association depends on pH-dependent electrostatic interactions [18,41]. Accordingly, the different precipitation behaviors may reflect changes in gelatin–lipid interactions and the balance of attractive and repulsive forces within the dispersion. However, this explanation remains hypothetical because particle size and zeta potential were not measured at each pH. Following CS coating, no visible precipitation was observed across the tested pH range. This improved stability can be attributed to the pH tolerance of CS and its protective coating effect, which prevents membrane aggregation and precipitation under acidic conditions [42,43]. The slightly greater turbidity observed for CS-GSH/GL is consistent with changes in particle size and surface composition following polymer coating [44]. These results demonstrate that CS coating effectively improved the stability of gelatin-containing liposomes under the tested pH range.

**Figure 4:**
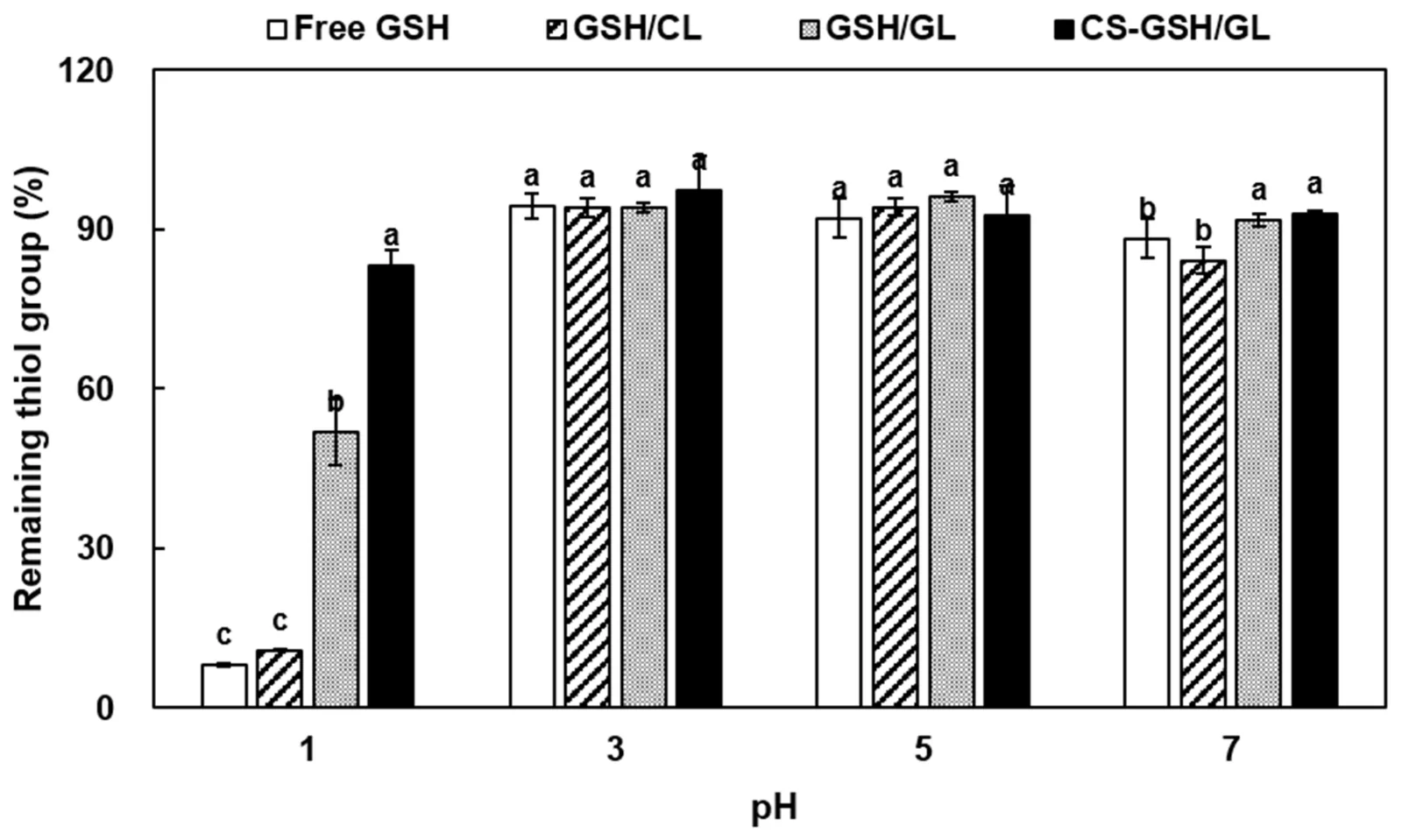
Effect of pH on the thiol group content of free GSH, GSH/CL, GSH/GL, and CS-GSH/GL. Different lowercase letters indicate significant differences (*p* < 0.05). Data are expressed as mean ± SD.

The changes in thiol group contents of free GSH and GSH-loaded liposomes under different pH conditions are presented in Figure 4. No significant changes were observed at pH 3 and pH 5 for any sample. However, at pH 1 and pH 7, free GSH and GSH/CL showed significantly reduced thiol contents (*p* < 0.05). In contrast, GSH/GL and CS-GSH/GL maintained significantly higher thiol levels, and CS-GSH/GL showed approximately 10.2-fold higher thiol content than free GSH at pH 1 (*p* < 0.05). Under strongly acidic conditions, characterized by a high concentration of hydrogen ions, peptides undergo hydrolysis [33,45]. Although most interactions involving thiol groups in GSH molecules are covalent, hydrogen bonding has also been reported [46]. Therefore, a high hydrogen ion concentration may potentially affect these hydrogen-bonding interactions. In addition, under conditions with a low hydrogen ion concentration, such as pH 7 or above, thiol groups may undergo oxidation to form intra- and intermolecular disulfide bonds, resulting in a decrease in free thiol content [32]. These factors may contribute to the pH-dependent decreases in free thiol content observed at pH 1 and pH 7. The poor protection observed in GSH/CL suggests that conventional liposomes were insufficient to prevent leakage or degradation under extreme pH conditions [36]. By contrast, gelatin incorporation likely improved thiol stability by increasing internal viscosity and limiting diffusion [31]. In addition, CS coating may have further protected GSH by forming a surface barrier that increased membrane rigidity and improved resistance to acidic conditions [37,42,47]. These results demonstrate that core gelatinization combined with CS coating effectively preserved the thiol groups of GSH across a broad pH range.

### 3.4. In Vitro Release Property

**Figure 5:**
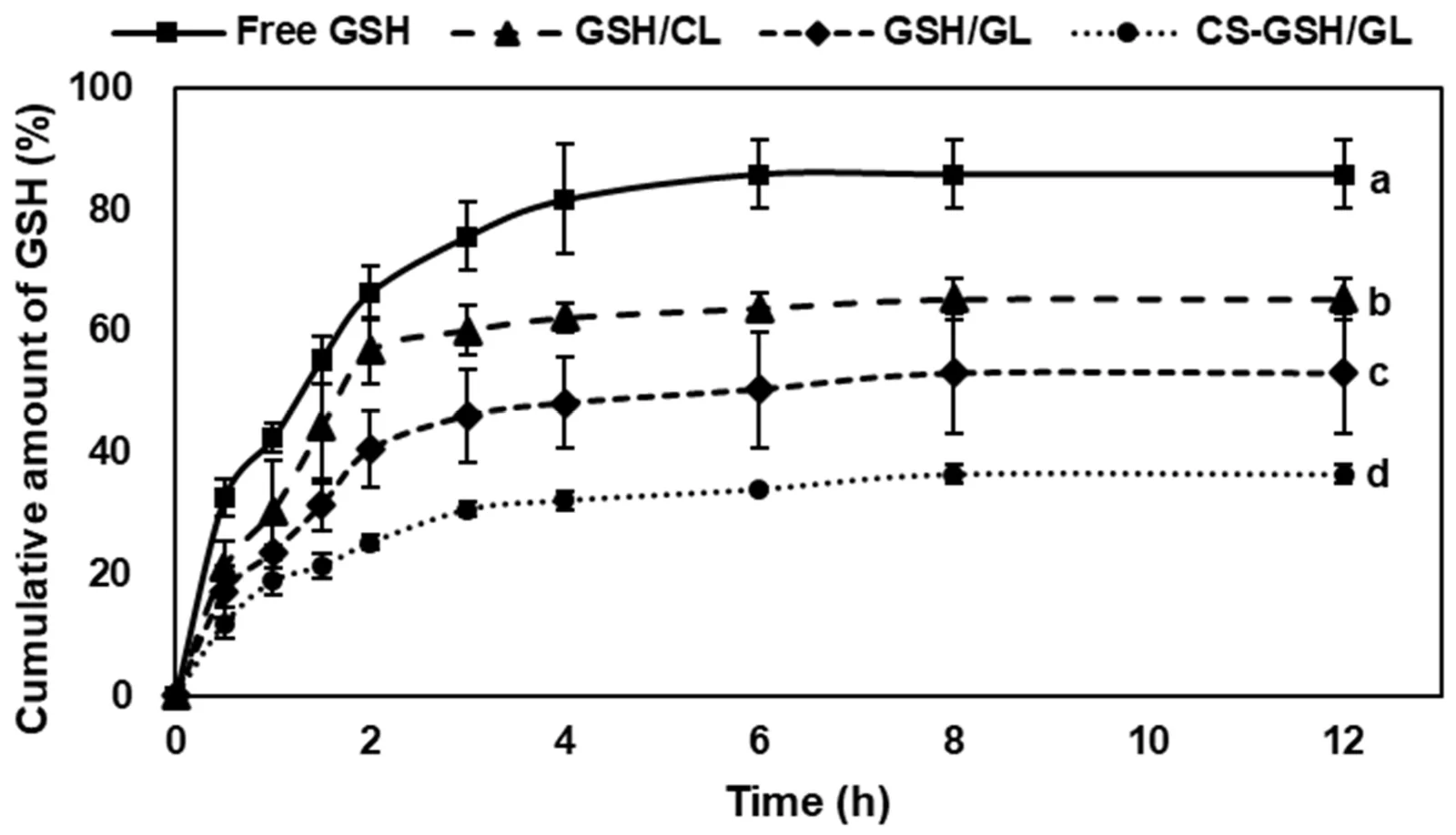
In vitro release profiles of free GSH, GSH/CL, GSH/GL, and CS-GSH/GL in SGF (pH 1.2) and SIF (pH 6.8) for 12 h. Different lowercase letters represent significant differences (*p* < 0.05). Data are expressed as mean ± SD.

The in vitro release profiles of free GSH and GSH-loaded liposomes under simulated gastrointestinal conditions are presented in Figure 5. Free GSH showed a rapid initial release of approximately 66% in SGF within 2 h, followed by further release in SIF, reaching a cumulative release of 86% after 12 h (*p* < 0.05). In contrast, GSH/CL, GSH/GL, and CS-GSH/GL showed significantly lower cumulative release rates of approximately 66%, 54%, and 36%, respectively, indicating sustained release behavior (*p* < 0.05). Notably, CS-GSH/GL exhibited the lowest cumulative release among all formulations (*p* < 0.05). The initial burst release of free GSH may be attributed to its unencapsulated fraction and its inherent susceptibility to rapid diffusion [48]. Although liposomal bilayers provide a protective barrier, prolonged exposure to gastrointestinal conditions can increase membrane permeability and facilitate drug leakage [36]. Gelatinized core liposomes improved retention of GSH by increasing core viscosity and reducing molecular diffusion [31]. Accordingly, GSH/GL and CS-GSH/GL showed lower release rates than conventional liposomes. In addition, CS coating likely enhanced diffusion resistance and membrane rigidity, further delaying GSH release [47,49]. The interaction between gelatin and polysaccharides has been shown to involve hydrogen bonding, electrostatic interactions, and ionic bonds, which collectively influence gel network structure and water mobility [50]. In the present system, similar interactions between the gelatinized core and the CS coating may contribute to the improved structural stability and retention observed for CS-GSH/GL. Collectively, these results indicate that gelatin incorporation and CS coating improved retention and sustained release of GSH under gastrointestinal conditions. However, the simulated gastrointestinal fluids used in this study did not contain digestive enzymes or bile salts. Therefore, the release profiles primarily reflect the effects of pH and ionic strength and do not account for the potential enzymatic degradation of the liposomal membrane and GSH.

### 3.5. Cytotoxicity

**Figure 6:**
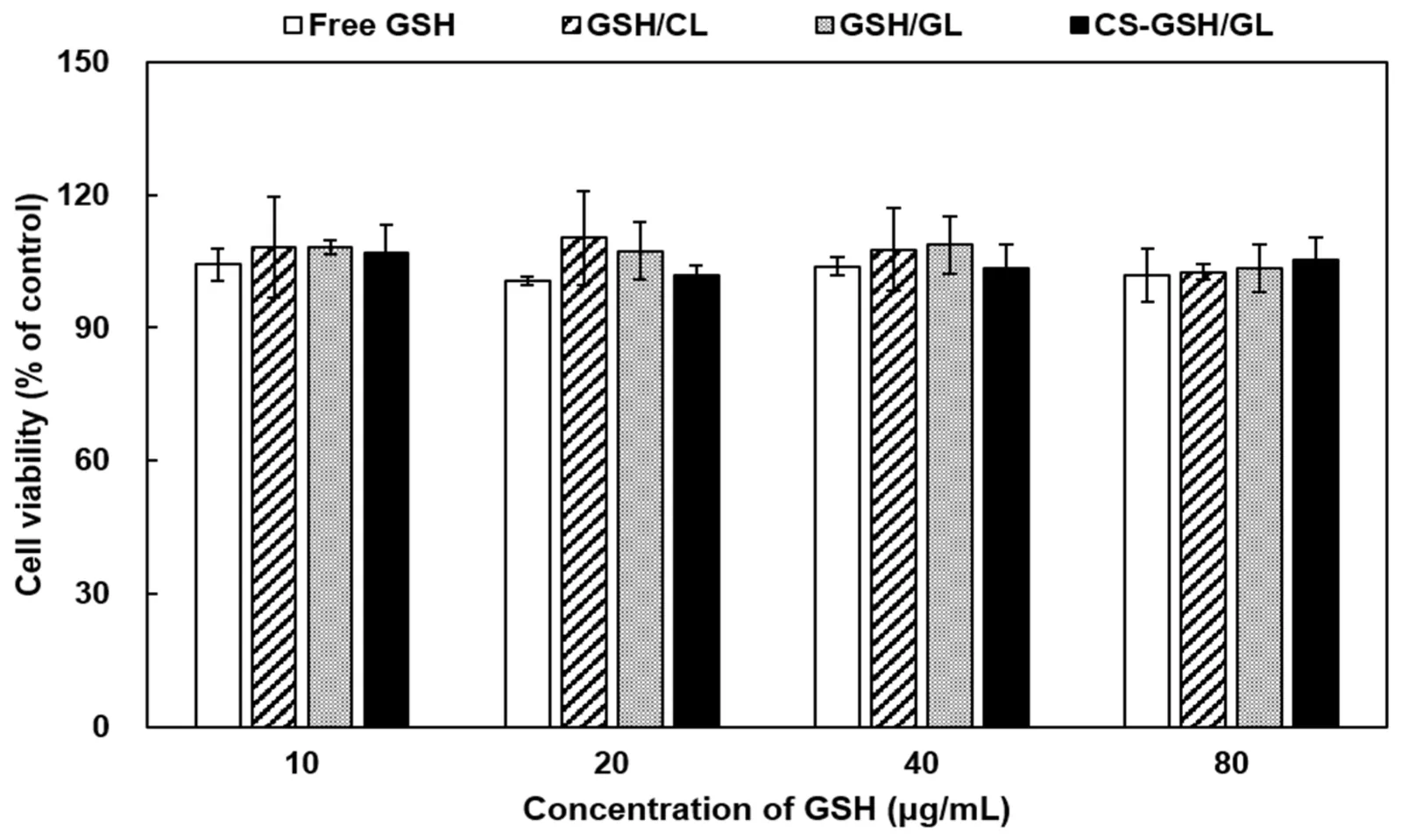
Cytotoxicity of free GSH, GSH/CL, GSH/GL, and CS-GSH/GL on Caco-2 cells. Data are expressed as mean ± SD.

The cytotoxicity of free GSH and GSH-loaded liposomes was evaluated in Caco-2 cells, and the results are presented in Figure 6. All samples exhibited cell viability above 90% over the tested concentration range of 10–80 µg/mL, with no significant differences among the formulations. Cell viability above 80% is generally considered indicative of non-cytotoxicity [51]. Thus, both free GSH and GSH-loaded liposomes were biocompatible in Caco-2 cells. These findings are consistent with previous reports showing that GSH-loaded nanocarriers do not induce significant cytotoxicity within similar concentration ranges [4,7].

### 3.6. Cellular Uptake

**Figure 7:**
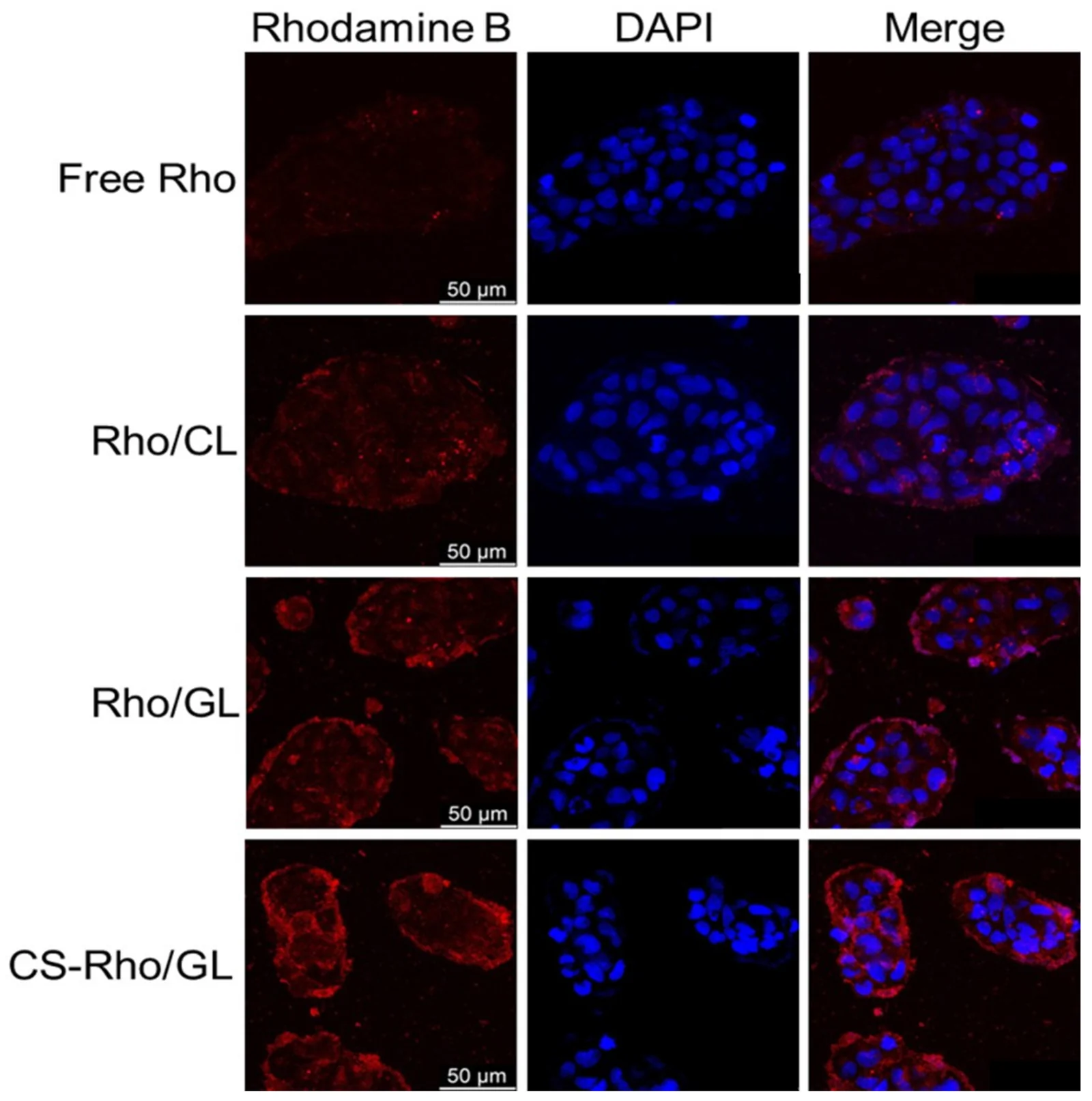
Cellular uptake of free Rho and Rho-loaded liposomes in Caco-2 cells. Confocal microscope images of Caco-2 cells incubated with free Rho, Rho/CL, Rho/GL, and CS-Rho/GL. Scale bar = 50 µm.

**Figure 8:**
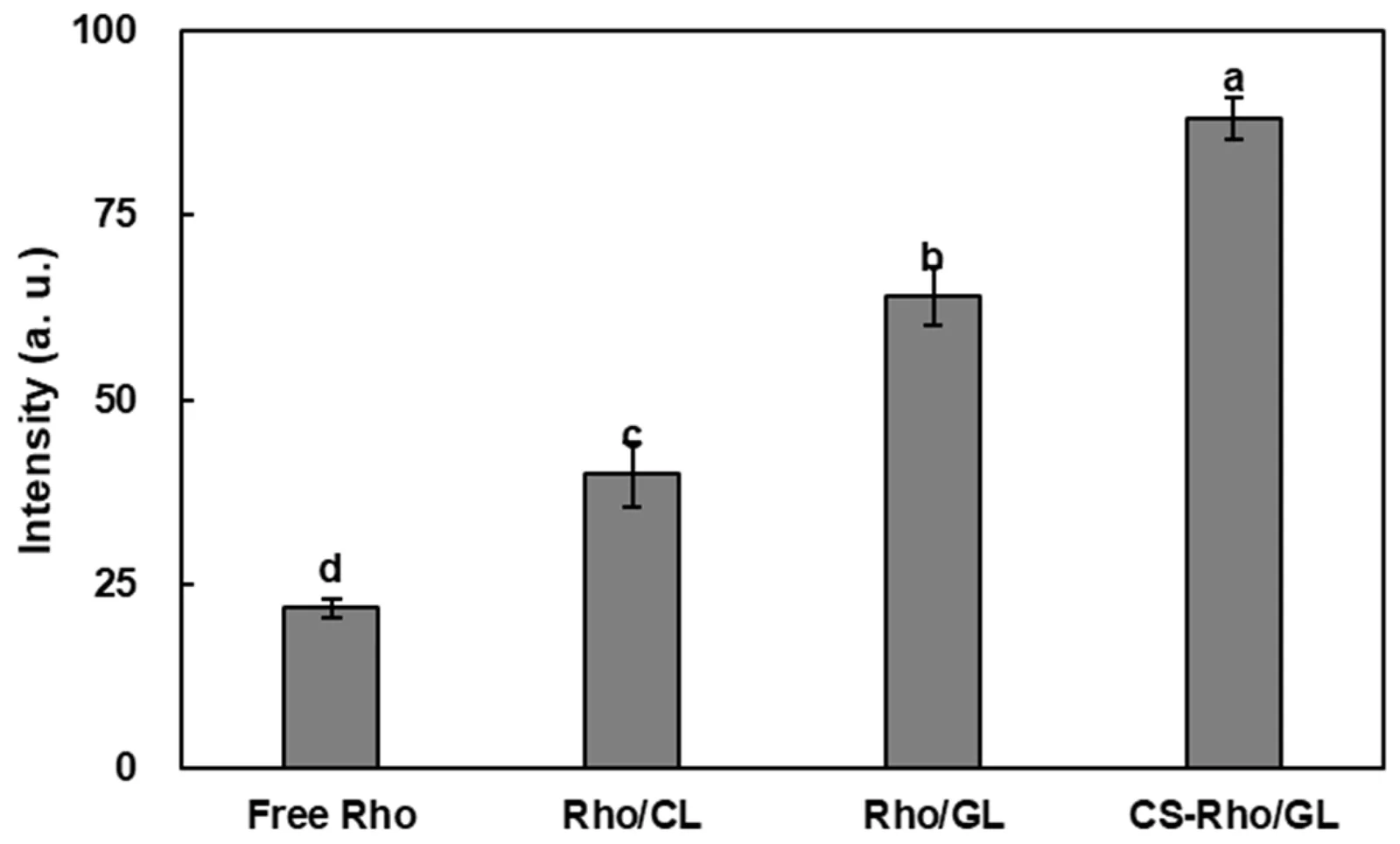
Quantification of intracellular Rho-associated fluorescence intensity using Las X software. Different lowercase letters represent significant differences (*p* < 0.05). Data are expressed as mean ± SD.

The effects of core gelatinization and CS coating on the cellular interaction of the liposomal formulations were evaluated in Caco-2 cells using rhodamine B (Rho) as a fluorescent model compound (Figure 7). The nuclei were stained with DAPI (blue fluorescence), while intracellular Rho-associated fluorescence was observed as red fluorescence. All liposomal formulations showed stronger intracellular fluorescence than free Rho, indicating greater cellular association of the fluorescently labeled formulations. Quantitative analysis of mean fluorescence intensity revealed a significant increase in the order of free Rho, Rho/CL, Rho/GL, and CS-Rho/GL (*p* < 0.05), with fluorescence intensity values of 21.81, 39.93, 63.96, and 88.14 a.u., respectively (Figure 8). CS-Rho/GL showed approximately 4.0-fold higher fluorescence intensity than free Rho, representing the highest fluorescence signal among the tested formulations (*p* < 0.05). The increased cellular fluorescence observed for the liposomal formulations may be associated with interactions between the lipid vesicles and the cell membrane, which can facilitate cellular association or vesicle-mediated internalization [52]. Gelatin incorporation may have contributed to the increased cellular association through changes in the physicochemical properties of the liposomes and their interactions with the cell surface [53]. CS coating further increased the fluorescence signal, which may be related to the positively charged surface of the CS-coated liposomes and its potential to promote interactions with negatively charged cell membranes [54]. Overall, the higher fluorescence intensity observed for CS-Rho/GL suggests that the combined core gelatinization and CS coating enhanced the cellular association of the liposomal formulation. Because Rho was used as a fluorescent model compound rather than GSH itself, these findings demonstrate the cellular association behavior of the modified liposomal system rather than direct intracellular uptake of GSH.

## 4. Conclusions

This study developed a biopolymer-stabilized liposomal system for GSH delivery by combining gelatin incorporation into the aqueous core with low-molecular-weight chitosan (CS) coating. Gelatin incorporation increased the entrapment efficiency of GSH compared with conventional liposomes, while subsequent CS coating further increased the entrapment efficiency and modified the surface charge of the liposomes. The CS-coated formulation also showed improved visible dispersion stability under acidic conditions and greater retention of thiol groups under the oxidative and pH conditions examined in this study. Under simulated gastrointestinal conditions, the modified liposomal formulations exhibited lower GSH release than free GSH, with CS-GSH/GL showing the lowest cumulative release among the tested formulations. These results indicate that gelatin incorporation and CS coating contributed to greater retention of GSH within the liposomal structure. In Caco-2 cells, the CS-coated gelatinized-core formulation showed the highest fluorescence intensity when Rho was used as a fluorescent model compound, indicating enhanced cellular association of the modified liposomal system. Overall, the combined use of gelatinized-core structuring and CS surface coating improved the physicochemical stability, GSH retention of liposomal formulation, while enhancing the cellular association of the Rho-loaded delivery system. These in vitro findings support the potential of CS-GSH/GL for GSH protection and retention. However, direct intracellular GSH uptake, intestinal absorption, and in vivo bioavailability were not evaluated. Further studies are therefore needed to establish the formulation’s suitability for oral GSH delivery.

## Figures and Tables

**Table 1:** The size, PDI, zeta potential, and entrapment efficiency of GSH/CL and GSH/GL.

	Physical Properties			
Samples	Particle Size (nm)	PDI	Zeta Potential (mV)	Entrapment Efficiency (%)
GSH/CL	100 ± 2 ^a^	0.260 ± 0.016 ^a^	−55 ± 4 ^b^	34 ± 6 ^b^
GSH/GL	92 ± 2 ^b^	0.241 ± 0.009 ^a^	−22 ± 1 ^a^	47 ± 3 ^a^

^a,b^ Different lowercase letters represent a significant difference (*p* < 0.05). Data are expressed as the mean value ± SD.

## Data Availability

The original contributions presented in this study are included in the article. Further inquiries can be directed to the corresponding author.
